# Dysglycemia and increased left ventricle mass in normotensive patients admitted with a first myocardial infarction: prognostic implications of dysglycemia during 14 years of follow-up

**DOI:** 10.1186/s12872-019-1084-5

**Published:** 2019-05-02

**Authors:** Gokulan Pararajasingam, Brian Bridal Løgstrup, Dan Eik Høfsten, Thomas Brøcher Christophersen, Søren Auscher, Jørgen Hangaard, Kenneth Egstrup

**Affiliations:** 1 Cardiovascular Research Unit, Odense University Hospital Svendborg, Baagøes Allé 15, 5700 Svendborg, Denmark; 2 Department of Cardiology, Aarhus University Hospital Skejby, Palle Juul Jensens Boulevard 99, 8200 Aarhus, Denmark; 3 Department of Cardiology, Copenhagen University Hospital Rigshospitalet, Blegdamsvej 9, 2100 Copenhagen, Denmark; 4 Department of Internal Medicine, Odense University Hospital Svendborg, Baagøes Allé 15, 5700 Svendborg, Denmark

**Keywords:** Dysglycemia, Oral glucose tolerance test, Left ventricle mass, Normotensive, Diabetes, Major adverse cardiovascular events, All-cause mortality

## Abstract

**Background:**

Left ventricle mass (LVM) can be influenced by various conditions including hypertension and/or inherent cardiomyopathies. Dysglycemia is also thought to exert an anabolic effect on heart tissue by hyperinsulinemia and thereby promoting increased LVM. The primary aim of this study was to assess the influence of dysglycemia on LVM evaluated by an oral glucose tolerance test (OGTT) in patients admitted with a first myocardial infarction (MI) without hypertension. The secondary aim was to assess the impact of dysglycemia on major adverse cardiovascular events (MACE) and all-cause mortality during long-term follow-up.

**Methods:**

Patients admitted with a first MI without known history of hypertension were included. All patients without previously known type 2 diabetes mellitus (T2DM) had a standardized 2-hour OGTT performed and were categorized as: normal glucose tolerance (NGT), impaired fasting glucose (IFG)/impaired glucose tolerance (IGT) and newly detected T2DM (new T2DM). LVM was measured by echocardiography using Devereaux formula and indexed by body surface area. Multivariate linear regression analysis was used to assess the impact of confounders (dysglycemia by OGTT, known T2DM, age, sex and type of MI) on LVM. Cox proportional hazard model was used to assess the impact of dysglycemia on all-cause mortality and a composite endpoint of MACE (all-cause mortality, MI, revascularisation due to stable angina, coronary artery bypass graft, ischemic stroke or hemorrhagic stroke).

**Results:**

Two-hundred-and-five patients were included and followed up to 14 years. In multivariate regression analysis, LVM was only significantly increased in patients categorized as new T2DM (β = 25.3; 95% CI [7.5–43.0]) and known T2DM (β = 37.3; 95% CI [10.0-64.5]) compared to patients with NGT. Patients with new T2DM showed higher rates of MACE and all-cause mortality compared to patients with IFG/IGT and NGT; however no significantly increased hazard ratio was detected.

**Conclusions:**

Dysglycemia is associated with increasing LVM in normotensive patients with a first acute myocardial infarction and the strongest association was observed in patients with new T2DM and patients with known T2DM. Dysglycemia in normotensive patients with a first MI is not an independent predictor of neither MACE nor all-cause mortality during long-term follow-up compared to normotensive patients without dysglycemia.

**Electronic supplementary material:**

The online version of this article (10.1186/s12872-019-1084-5) contains supplementary material, which is available to authorized users.

## Background

Increased left ventricle mass (LVM) is a well-known independent risk factor of adverse cardiovascular events [1, 2]. The effect of increased LVM on long-term outcomes in patients with ST-segment elevation myocardial infarction (MI) has been investigated [3, 4]. Increased LVM is associated with increased all-cause mortality [1, 2] and recurrent MI [4]. Hypertension [5] and aortic valve stenosis [6] are well-known causes of increased LVM, but dysglycemia has also been thought to influence LVM [7, 8]. The prevalence of dysglycemia is increasing worldwide and is a well-known risk factor of cardiovascular disease, which increases morbidity and mortality [9, 10]. Patients with overt dysglycemia frequently deal with concomitant conditions such as hypertension as a part of the metabolic syndrome (MetS) [11]. Santra et al. [12] found significantly increased indexed LVM in normotensive patients with type 2 diabetes mellitus (T2DM) compared to a group of normotensive age-and-sex matched non-diabetic patients. Another study by Sciacqua et al. [13] investigated dysglycemia by a 2-hour oral glucose tolerance test (OGTT) in hypertensive patients. They found a direct correlation between 1-hour post load glucose values and LVM. To our knowledge, no studies have specifically investigated the effect of dysglycemia assessed by OGTT on LVM in patients with first MI without a history of hypertension.

Our primary aim was to assess the influence of dysglycemia evaluated by an OGTT on LVM in a population admitted with a first MI without hypertension. Our secondary aim was to investigate the impact of dysglycemia evaluated by an OGTT on major adverse cardiovascular events (MACE) and all-cause mortality during long-term follow-up.

## Methods

Inclusion criteria were: age ≥ 18 years, written informed consent, MI according to contemporary guidelines according to European recommendations based on documented elevation of Troponin-T > 0.1 μg/L and characteristic symptoms of MI and/or electrocardiographic signs of MI [14]. Inclusion criterion for this specific study was a MI.

Exclusion criteria were previous MI, history of hypertension, missing data on OGTT in patients without diabetes, missing data on LVM, usage of any antihypertensive medication and patients with type 1 diabetes.

### Collection of data

This study was a single centre study based on data collected from 4 prior studies conducted on patients admitted with a MI at Odense University Hospital Svendborg from august 2004 until august 2013 [15–18]. This study had an observational design (primary aim) but also a prospective design (secondary aim). All patients were consecutively included into their respective study. Data on cardiac interventional procedures was collected through the Western Danish Heart Registry, which is a clinical database containing all cardiac invasive procedures in western part of Denmark. Data on ischemic stroke and hemorrhagic stroke was obtained by electronic medical record system and adjudicated according to treating physician. Data on all-cause mortality was collected through our regional medical record system, which is directly linked to the national civil registration system.

### Usage of data

This study analysed specific data such as age, height, weight, sex, left ventricle mass, left ventricle ejection fraction (LVEF), left ventricle end diastolic dimension, posterior wall end diastolic dimension, interventricular septum end diastolic dimension, glucometabolic status according to OGTT, known T2DM, fasting glucose, glycosylated hemoglobin A1c (HbA1c), cholesterol and triglyceride levels, non-ST-segment elevation myocardial infarction (NSTEMI) and ST-segment elevation myocardial infarction (STEMI), stable angina, coronary artery bypass graft (CABG), ischemic stroke, hemorrhagic stroke, all-cause mortality, antihypertensive medication and antidiabetic medication.

### Ethics

Relevant permissions were obtained from both the National Board of Health (Danish Patient Safety Authority) and the Danish Data Agency (Region of Southern Denmark).

### Assessment of glucometabolic status

An OGTT was performed after an overnight fast in patients without a history of T2DM. Patients with T2DM were classified by self-reporting along with regular antidiabetic medication. Capillary whole blood and venous blood sample were drawn, followed by the administration of 75 g glucose solution. Blood glucose level was measured again after 2 hour. Capillary whole blood was assessed using a Hemocue 201+ glucose analyser. Patients were grouped according to 1999 WHO criteria for capillary whole blood and venous blood glucose levels in mmol/l [19]. Capillary whole blood sample was drawn in three studies (BBL, DEH and TBC), whereas venous blood sample was drawn in one study (SA). Dysglycemia was defined as patients with an abnormal glucose tolerance test or known T2DM.

#### Capillary whole blood

Normal glucose tolerance (NGT) was defined as a fasting whole blood capillary glucose concentration < 5.6 mmol/l and 2-hour post-load glucose concentration < 7.8 mmol/l. Impaired fasting glycemia (IFG) was defined as a fasting glucose concentration ≥ 5.6 mmol/l and < 6.1 mmol/l and a 2-hour post-load glucose concentration < 7.8 mmol/l. Impaired glucose tolerance (IGT) was defined as a fasting glucose concentration < 6.1 mmol/l and a 2-hour post-load glucose concentration ≥ 7.8 mmol/l and < 11.0 mmol/l. Newly detected T2DM (new T2DM) was defined by a fasting glucose concentration ≥ 6.1 mmol/l or a 2-hour glucose post-load concentration ≥ 11.1 mmol/l. Abnormal glucose tolerance (AGT) was defined as IFG/IGT and new T2DM.

#### Venous blood

NGT was defined as a fasting venous blood glucose concentration < 6.1 mmol/l and 2-hour post-load glucose concentration < 7.8 mmol/l. IFG was defined as a fasting glucose concentration ≥ 6.1 mmol/l and < 7.0 mmol/l and a 2-hour post-load glucose concentration < 7.8 mmol/l. IGT was defined as a fasting glucose concentration < 7.0 mmol/l and a 2-hour post-load glucose concentration ≥ 7.8 mmol/l < 11.0 mmol/l. New T2DM was defined by a fasting glucose concentration ≥ 7.0 mmol/l or a 2-hour glucose post-load concentration ≥ 11.1 mmol/l. Abnormal glucose tolerance (AGT) was defined as IFG/IGT and new T2DM.

### Echocardiography

Transthoracic echocardiography was performed during index hospitalization for MI using available ultrasound systems from 2 different vendors (Philips & GE). All images were analysed offline by a single investigator in the original study, blinded to all clinical data. LVM was calculated from Devereux standard formula (0.8*(1.04*(left ventricle end diastolic dimension + posterior wall end diastolic dimension + interventricular septum end diastolic dimension) ^ 3 - (left ventricle end diastolic dimension) ^ 3) + 0.6) and indexed. Indexation was done by body surface area (BSA) using Du Bois formula (0.007184 * weight ^ 0.425 * height ^ 0.725). Limits for left ventricle hypertrophy (LVH) were considered as 116 g/m^2^ for men and 104 g/m^2^ for women [20]. LVEF was determined by Simpson’s biplane. Early transmitral flow velocity to atrial transmitral flow velocity ratio (E/A ratio) was obtained from apical 4 chamber view.

### Hypertension

Patients with hypertension were classified by self-reporting and/or use of regular antihypertensive medication (angiotensin-converting-enzyme, angiotensin-receptor-II-antagonist, calcium-antagonist, beta-blocker or diuretics). Patients with elevated blood pressure during index admission were only considered as patients with hypertension if any antihypertensive medication was initiated.

### Major adverse cardiovascular events

A composite endpoint of events (ischemic stroke, hemorrhagic stroke, all-cause mortality, CABG, revascularization due to a myocardial infarction or stable angina) were detected after admission with index MI and registered as MACE. Patients with multiple cardiovascular events were only registered with a composite endpoint of MACE once, whereas the date of the first MACE was used for statistical analysis. End of follow-up for MACE and all-cause mortality was October 25, 2018.

### Statistical analysis

Descriptive statistics included counts and percentages for categorical variables, whereas continuous statistics were presented as mean and standard deviation (SD). Continuous variables were compared using one-way ANOVA, while categorical variables were compared using χ^2^. Spearman Rank correlation was used to assess the correlation between the degree of dysglycemia and LVM. Results were reported with β-coefficients in multivariate linear regression model with an associated 95% confidence interval (CI). Log rank test was used for comparing the differences in Kaplan-Meier curve. Results were reported with Hazard Ratios (HR) in cox proportional hazard model along with an associated 95% CI. Assumptions of proportionality and linearity were checked and found satisfactory. A two-sided *p*-value of < 0.05 was considered statistically significant in all statistic calculations. All analyses were performed with STATA.

### Linear regression analysis

Four different multivariate linear regression models were used to analyse the effect of glucometabolic status on LVM. Glucometabolic status was evaluated by OGTT in patients without known T2DM as a categorical variable (NGT, IFG/IGT and new T2DM) but also as a modified categorical variable (NGT and AGT). Glucometabolic status was furthermore evaluated by HbA1c and fasting glucose as continuous variables in patients without known T2DM. All statistical linear regression models included multivariate adjustment for confounders as following: age, sex and type of infarction (STEMI/NSTEMI). They were chosen prior to statistical analysis due to potential confounding of LVM.

### Cox proportional hazard model

Survival analysis was used to analyse the effect of dysglycemia by OGTT (NGT, IFG/IGT, new T2DM) and known DM on both MACE and all-cause mortality. The number of confounders for adjustment in the multivariate regression models was according to “the rule of ten” (maximum 10% of the total number of events). The natural order of confounders applied to the cox proportional hazard model was as following: the degree of dysglycemia, sex, type of infarction and age.

## Results

Baseline characteristics for the final study population are shown in Table 1. The total study population consisted of 205 patients and is shown in the STROBE diagram [see Additional file 1] and were followed up to 14 years with a mean follow-up time of 10 years.

**Table 1:** Baseline characteristics of 205 patients stratified by a 2-hour OGTT

	NGT	IFG/IGT	New T2DM	Known T2DM	P
Patients, n (%)	85 (41)	70 (34)	38 (19)	12 (6)	
Age (years)	60 ± 12	60 ± 13	65 ± 11	63 ± 8	NS
Follow-up (years)	10 ± 3	10 ± 3	10 ± 4	11 ± 2	NS
Men, n (%)	72 (85)	59 (84)	23 (61)	10 (83)	0.04
STEMI, n (%)	50 (59)	43 (61)	22 (58)	8 (67)	NS
SBP (mmHg)	139 ± 27	136 ± 26	143 ± 30	119 ± 25	NS
DBP (mmHg)	86 ± 20	84 ± 18	81 ± 16	76 ± 19	NS
BMI (g/m^2^)	26 ± 5	27 ± 4	26 ± 5	28 ± 5	NS
Current Smoker, n (%)	47 (55)	46 (66)	20 (53)	5 (42)	NS
Medication:					
ASA, n (%)	12 (14)	8 (11)	5 (13)	2 (18)	NS
Statins, n (%)	66 (78)	60 (86)	32 (84)	10 (83)	NS
Biguanides, n (%)	0 (0)	0 (0)	0 (0)	3 (27)	N/A
Insulin, n (%)	0 (0)	0 (0)	0 (0)	4 (36)	N/A
Blood samples:					
HbA1c (%)	5.5 ± 0.3	5.6 ± 0.4	6.0 ± 1.2	7.6 ± 1.4	< 0.001
Fasting glucose (mmol/l)	4.7 ± 0,5	5.1 ± 0.6	6.2 ± 1.9	N/A	< 0.001
2-hour glucose (mmol/l)	6.3 ± 1.1	8.6 ± 1.0	11.7 ± 2.9	N/A	< 0.001
LDL (mmol/L)	3.1 ± 1.0	2.8 ± 0.8	2.5 ± 0.8	2.4 ± 0.6	0.01
HDL (mmol/L)	1.2 ± 0.4	1.1 ± 0.3	1.1 ± 0.4	1.1 ± 0.3	NS
Total cholesterol (mmol/L)	5.0 ± 1.2	4.7 ± 1.0	4.3 ± 1.0	4.0 ± 0.8	0.01
Triglyceride (mmol/L)	1.5 ± 0.9	1.6 ± 1.0	1.5 ± 0.8	1.3 ± 0.6	NS
Echocardiographic examination:					
LVM (g/m^2^)	102 ± 36	111 ± 42	123 ± 61	138 ± 56	0.02
LVH, n (%)	21 (25)	26 (37)	16 (42)	8 (67)	0.01
IVS (cm)	1.0 ± 0.2	1.1 ± 0.3	1.1 ± 0.3	1.2 ± 0.3	0.03
LVID (cm)	4.9 ± 0.7	5.0 ± 0.9	5.0 ± 0.8	5.1 ± 0.4	NS
LVPW (cm)	1.1 ± 0.3	1.1 ± 0.3	1.1 ± 0.3	1.2 ± 0.2	NS
LVEF (%)	52 ± 11	51 ± 12	50 ± 12	54 ± 8	NS
E/A ratio	1.2 ± 0.5	1.2 ± 0.7	1.0 ± 0.4	1.5 ± 1.4	NS

*OGTT* oral glucose tolerance test, *NGT* normal glucose tolerance, *IFG/IGT* impaired fasting glucose/impaired glucose tolerance, *New T2DM* newly detected type 2 diabetes mellitus, *Known T2DM* known type 2 diabetes mellitus, *STEMI* ST-segment elevation myocardial infarction, *SBP* systolic blood pressure, *DBP* diastolic blood pressure, *BMI* body mass index, *ASA* acetylsalicylic acid, *HbA1c* hemoglobin A1c, *LDL* low density lipoprotein, *HDL* high density lipoprotein, *LVM* left ventricle mass, *LVH* left ventricle hypertrophy, *IVS* inter ventricular septum thickness, *LVID* left ventricle internal diameter, *LVPW* left ventricle posterior wall thickness, *LVEF* left ventricle ejection fraction, *E/A ratio* early to late ventricular filling velocity ratio, *N/A* not applicable, *NS* not significant

One-hundred-and-sixty-four (80%) patients were men, mean age was 61 years and 123 (60%) patients were hospitalized with a STEMI. Median time for the performance of an OGTT after MI was 3 days [IQR 1–6] and 2 days for echocardiography [IQR 0–4].

Forty-six patients died during follow-up, whereas 39 (85%) patients had data on date of death available for cox proportional hazard model. Eighty-four events of MACE were registered, but 4 events of MACE occurred in the same patients, whereas 80 events of MACE were used for cox proportional hazard model.

### Baseline characteristics according to oral glucose tolerance test

The standardized 2-hour OGTT results categorized 85 (41%) patients as NGT, 70 (34%) patients were categorized as IFG/IGT and 38 (19%) patients as new T2DM. Twelve (6%) patients had known T2DM (Table 1). Age, follow-up time and type of MI did not differ across OGTT-groups. Sex differed across OGTT-groups. Cardiovascular risk factors such as smoking, body mass index (BMI) and blood pressure did not differ across OGTT-groups. No significant differences were observed regarding the cholesterol lowering and anticoagulation treatment. A significant decreasing trend was observed in total cholesterol across OGTT-groups, which was primarily driven by the significant decreasing trend in LDL cholesterol. HDL and triglycerides did not differ significantly. A significantly increasing trend was observed in HbA1c, fasting glucose and 2-hour glucose across OGTT-groups. Indexed LVM and interventricular septum (IVS) thickness showed a significant increasing trend across OGTT-groups, whereas no significant differences were observed in left ventricle internal diameter, left ventricle posterior wall thickness, LVEF and E/A ratio.

### Relation between dysglycemia and left ventricle mass

**Fig. 1:**
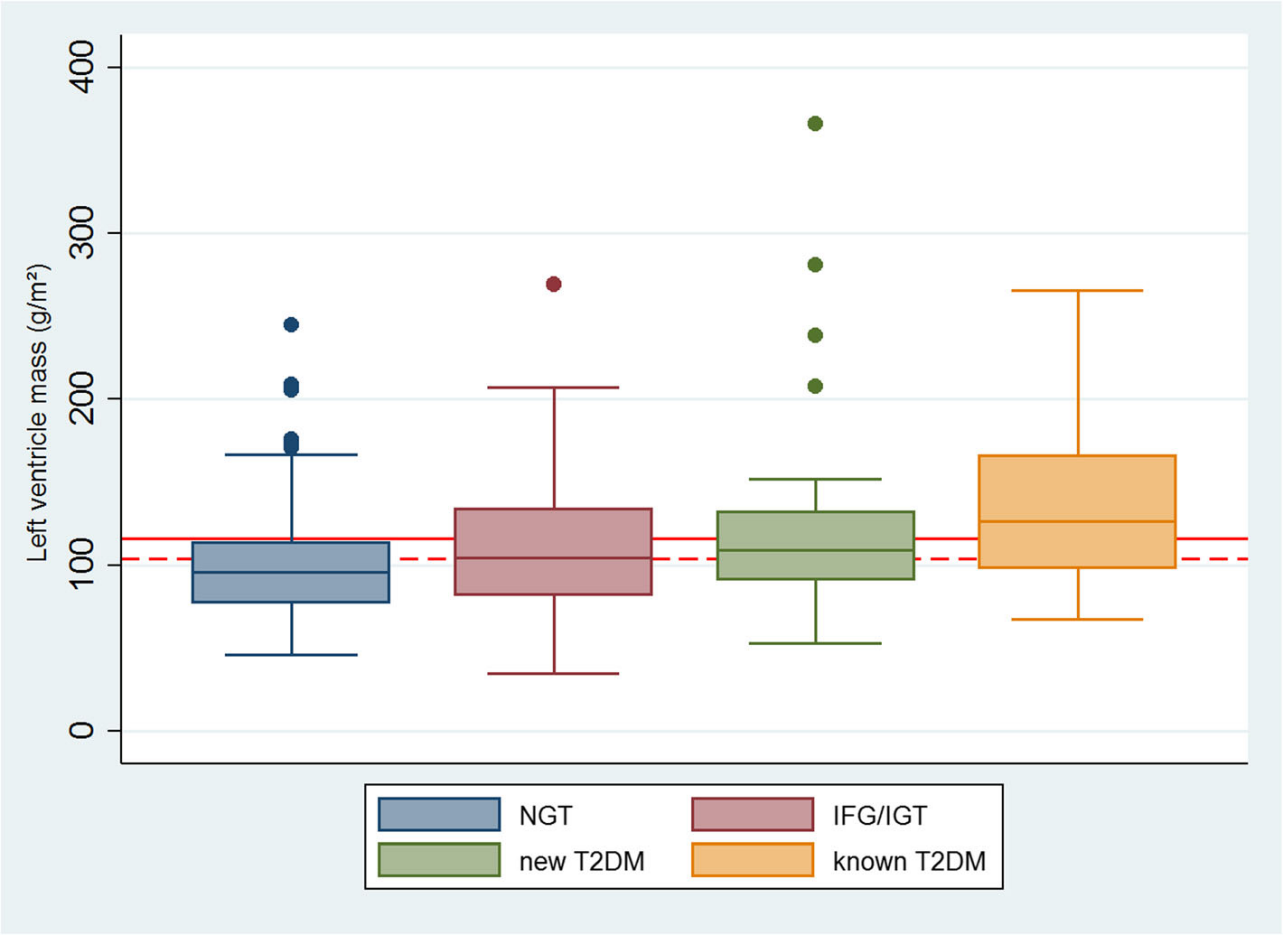
Indexed left ventricle mass stratified by an oral glucose tolerance test in 205 normotensive patients with a first myocardial infarction. Boxplot with median of indexed left ventricle mass and corresponding inter-quartile range. Limits for left ventricle hypertrophy for men (red solid line) and women (red dash line)

The boxplot (Fig. 1) showed the impact of dysglycemia on LVM (p-trend: 0.01; correlation coefficient 0.21). Increased LVM for both men and women was observed in 21 patients with NGT (25%), 26 patients with IFG/IGT (37%), 16 patients with new T2DM (42%) and 8 patients with known T2DM (67%).

In multivariate linear regression model A (age, sex, the degree of dysglycemia by OGTT (NGT, IFG/IGT, new T2DM), known T2DM and type of infarction) (Table 2A), LVM was significantly higher in patients categorized as new T2DM (β 25.3 [95% CI 7.5-43.0]) and known T2DM (β 37.3 [95% CI 10.0-64.5]) compared to patients categorized as NGT. LVM was not significantly increased in patients categorized as IFG/IGT (β 9.4 [95% CI -4.8-23.6]) compared to patients categorized as NGT. In addition, men had significantly higher LVM (β 17.6 [95% CI 1.4-33.8]) compared to women. Age did not impact LVM significantly (β -0.1 [95% CI -0.5-0.5]). Patients with STEMI did not have significantly lower LVM (β -6.3 [95% CI -19.0-6.4]) compared to patients with NSTEMI.

**Table 2:** Multivariate linear regression model on indexed LVM in patients in 205 patients

A	β	95% CI		P	B	β	95% CI		P
NGT (OGTT)	ref	ref	ref		NGT (OGTT)	ref	ref	ref	
IFG/IGT (OGTT)	9.4	-4.8	23.6	NS	AGT (OGTT)	14.7	1.8	27.6	0.03
New T2DM (OGTT)	25.3	7.5	43.0	0.01					
Known T2DM	37.3	10.0	64.5	0.01	Known T2DM	37.0	9.6	64.4	0.01
Women	ref	ref	ref		Women	ref	ref	ref	
Men	17.6	1.4	33.8	0.03	Men	15.2	-0.8	31.1	NS
NSTEMI	ref	ref	ref		NSTEMI	ref	ref	ref	
STEMI	- 6.3	-19.0	6.4	NS	STEMI	−6.3	−19.0	6.5	NS
Age	- 0.1	−0.5	0.5	NS	Age	0.1	−0.5	0.6	NS

*LVM* left ventricle mass, *OGTT* oral glucose tolerance test, *NGT* normal glucose tolerance, *IFG/IGT* impaired fasting glucose/impaired glucose tolerance, *New T2DM* newly detected type 2 diabetes mellitus, *Known T2DM* known type 2 diabetes mellitus, *AGT* abnormal glucose tolerance, *NSTEMI* non-ST-segment elevation myocardial infarction, *STEMI* ST-segment elevation myocardial infarction, *NS* not significant, *ref* reference

In multivariate linear regression model B (age, sex, the degree of dysglycemia by OGTT (NGT, AGT), known T2DM and type of infarction) (Table 2B), LVM was significantly higher in patients categorized as AGT (β 14.7 [95% CI 1.8-27.6]) and known T2DM (β 37.0 [95% CI 9.6-64.4]) compared to patients categorized as NGT. Men had higher LVM (β 15.2 [95% CI -0.8-31.1]) compared to women, however not statistically significant. Age did not impact LVM significantly (β 0.1 [95% CI -0.5-0.6]). Patients with STEMI did not have significantly lower LVM (β -6.3 [95% CI -19.0-6.5]) compared to patients with NSTEMI.

In multivariate linear regression model C (age, sex, HbA1c and type of infarction) (Table 3C), LVM was not significantly increased with one percentage point increase in HbA1c (β 5.8 [95% CI -4.2-15.8]). Men had significantly increased LVM (β 19.2 [95% CI 2.9-35.5]) compared to women. Age did not increase LVM significantly (β 0.1 [95% CI -0.5-0.6]). Patients with STEMI did not have significantly lower LVM compared to patients with NSTEMI (β -5.2 [95% CI -18.2-7.8]).

**Table 3:** Multivariate linear regression on indexed LVM in 193 patients without known T2DM and HbA1c (C) and Fasting glucose (D)

C^a^	β	95% CI		P	D	β	95% CI		P
HbA1c	5.8	−4.2	15.8	NS	Fasting glucose	7.6	1.9	13.3	0.01
Women	ref	ref	ref		Women	ref	ref	ref	
Men	19.2	2.9	35.5	0.02	Men	20.5	4.5	36.4	0.01
NSTEMI	ref	ref	ref		NSTEMI	ref	ref	ref	
STEMI	- 5.2	−18.2	7.8	NS	STEMI	−4.1	−16.9	8.7	NS
Age	0.1	−0.5	0.6	NS	Age	0.0	−0.5	0.6	NS

*LVM* left ventricle mass, *HbA1c* glycosylated hemoglobin A1c, *NSTEMI* non-ST-segment elevation myocardial infarction, *STEMI* ST-segment elevation myocardial infarction, *NS* not significant, *ref* reference

^a^192 had data on HbA1c

In multivariate linear regression model D (age, sex, fasting glucose and type of infarction) (Table 3d), LVM was significantly increased with one mmol/l increase in fasting glucose (β 7.6 [95% CI 1.9-13.3]). Men had significantly increased LVM compared to women (β 20.5 [95% CI 4.5-36.4]). Age did not increase LVM significantly (β 0.0 [95% CI -0.5-0.6]). Patients with STEMI did not have significantly lower LVM compared to patients with NSTEMI (β -4.1 [95% CI -16.9-8.7]).

### Relation between dysglycemia and major adverse cardiovascular events

There was no trend in events of MACE across OGTT-groups (Table 4). No significant differences were observed in the number of revascularisations, all-cause mortality or the number of strokes across OGTT-groups.

**Table 4:** Number of major adverse cardiovascular events in 205 patients stratified by a 2-hour OGTT

	NGT	IFG/IGT	New T2DM	Known T2DM	P
MACE^a^, n (%)	35 (41)	23 (33)	18 (47)	4 (33)	NS
All-cause mortality, n (%)	18 (21)	13 (19)	12 (32)	3 (25)	NS
Cardiac revascularisation (NSTEMI/STEMI/SA/CABG), n (%)	11 (13)	10 (14)	5 (13)	3 (25)	NS
Stroke (ischemic/hemorrhagic), n (%)	6 (7)	1 (1)	2 (5)	0 (0)	NS

*MACE* major adverse cardiovascular events, *OGTT* oral glucose tolerance test, *NGT* normal glucose tolerance, *IFG/IGT* impaired fasting glucose/impaired glucose tolerance, *New T2DM* newly detected type 2 diabetes mellitus, *Known T2DM* known type 2 diabetes mellitus, *NSTEMI* non-ST-segment elevation myocardial infarction, *STEMI* ST-segment elevation myocardial infarction, *SA* stable angina, *CABG* coronary bypass graft, *NS* not significant

^a^Patients with multiple cardiovascular events were only accounted for their first MACE

**Fig. 2:**
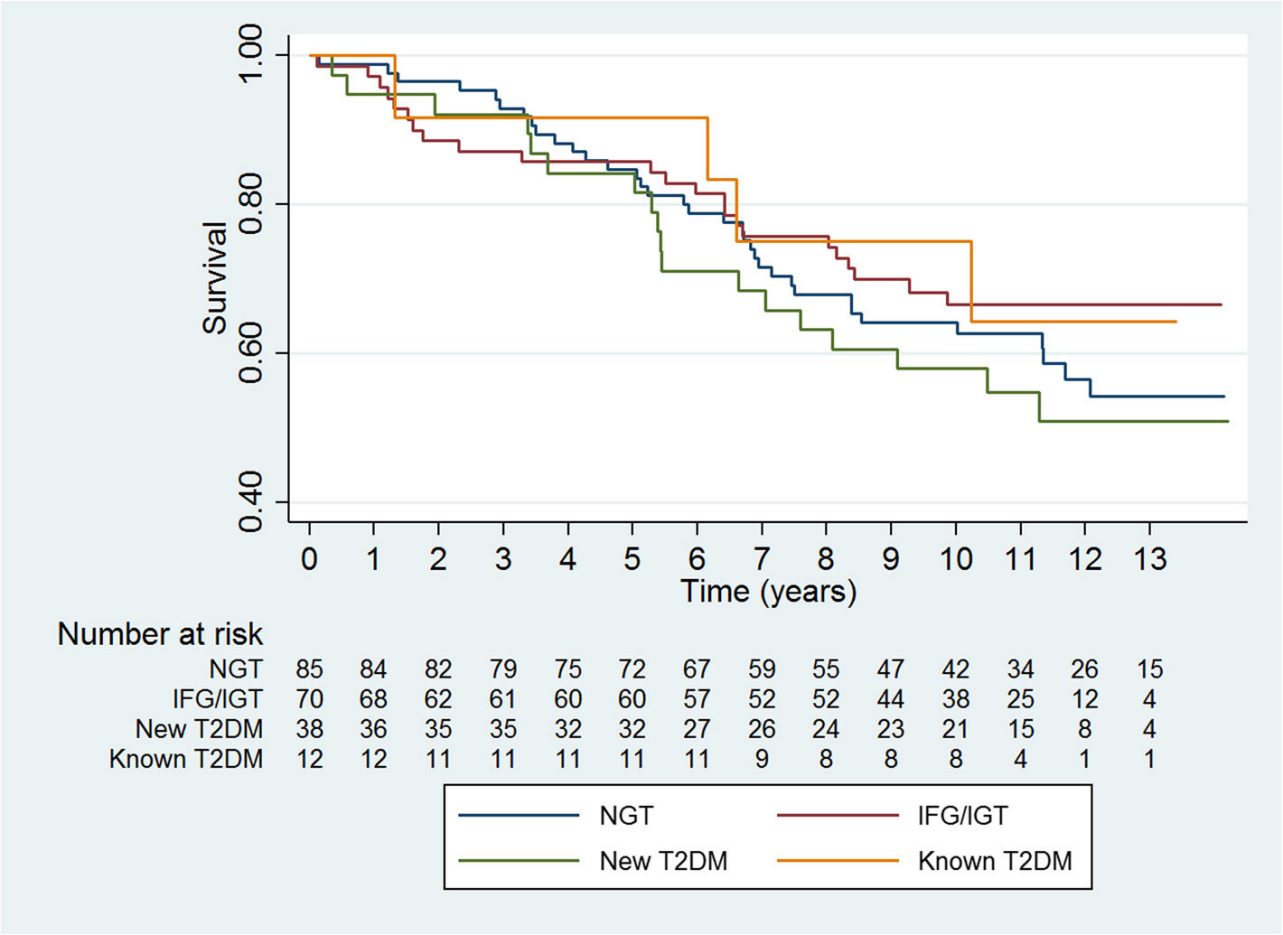
Kaplan-Meier curve shows survival according to major adverse cardiovascular events in 205 patients admitted with an acute myocardial infarction up to 14 years of follow-up. Patients without known type 2 diabetes mellitus were stratified according to an oral glucose tolerance test performed at time of admission

Patients were followed with a mean follow-up time of 9 years until first MACE. Kaplan-Meier curve showed (Fig. 2) event rates of MACE in relation to dysglycemia. Patients with new T2DM had the highest event rates of MACE compared to other OGTT-groups. Patients with IFG/IGT had lower event rates of MACE compared to patients with NGT, but comparable event rates of MACE as patients with known T2DM. Log rank test showed no difference between OGTT-groups (*P* = 0.59).

In cox proportional hazard regression model (glucometabolic status by OGTT (NGT, IFG/IGT, new T2DM) and known T2DM), higher HR of MACE was only observed in patients with new T2DM (HR 1.15 [95% CI 0.65-2.07]) compared to patients with NGT, however not statistically significant (Table 5E). Patients with IFG/IGT (HR 0.79 [95% CI 0.47-1.34]) and known T2DM (HR 0.78 [95% CI 0.28-2.21]) had lower HR of MACE compared to patients with NGT, however not statistically significant. Sex and type of infarction did not predict MACE independently.

**Table 5:** Cox proportional hazard regression model on MACE (E) and all-cause mortality (F) in 205 patients

E	HR	95% CI		P	F	HR	95% CI		P
NGT (OGTT)	ref	ref	ref		NGT (OGTT)	ref	ref	ref	
IFG/IGT (OGTT)	0.79	0.47	1.34	NS	IFG/IGT (OGTT)	0.91	0.41	1.99	NS
New T2DM (OGTT)	1.15	0.65	2.07	NS	New T2DM (OGTT)	1.93	0.90	4.12	NS
Known T2DM	0.78	0.28	2.21	NS	Known T2DM	0.47	0.06	3.54	NS
Women	ref	ref	ref						
Men	0.85	0.49	1.47	NS					
NSTEMI	ref	ref	ref						
STEMI	0.85	0.54	1.33	NS					

*MACE* major adverse cardiovascular event, *HR* hazard ratio, *NGT* normal glucose tolerance test, *IFG/IGT* impaired fasting glucose/impaired glucose tolerance, *New T2DM* newly detected type 2 diabetes mellitus, *Known T2DM* known type 2 diabetes mellitus, *OGTT* oral glucose tolerance test, *NSTEMI* non-ST-segment elevation myocardial infarction, *STEMI* ST-segment elevation myocardial infarction, *NS* not significant, *ref* reference

Log rank test showed no significant difference in patients with NGT and IFG/IGT (*P* = 0.41). Patients with NGT and IFG/IGT were then pooled and used in a modified cox proportional hazard regression model. Patients with new T2DM (HR 1.26 [95% CI 0.73-2.20]) showed an increased HR compared to patients with NGT/IFG/IGT; however this was not statistically significant. Patients with known T2DM (HR 0.87 [95% CI 0.31-2.39]) showed an insignificantly reduced HR compared to patients with NGT/IFG/IGT. Sex and type of infarction did not predict MACE independently.

### Relation between dysglycemia and all-cause mortality

**Fig. 3:**
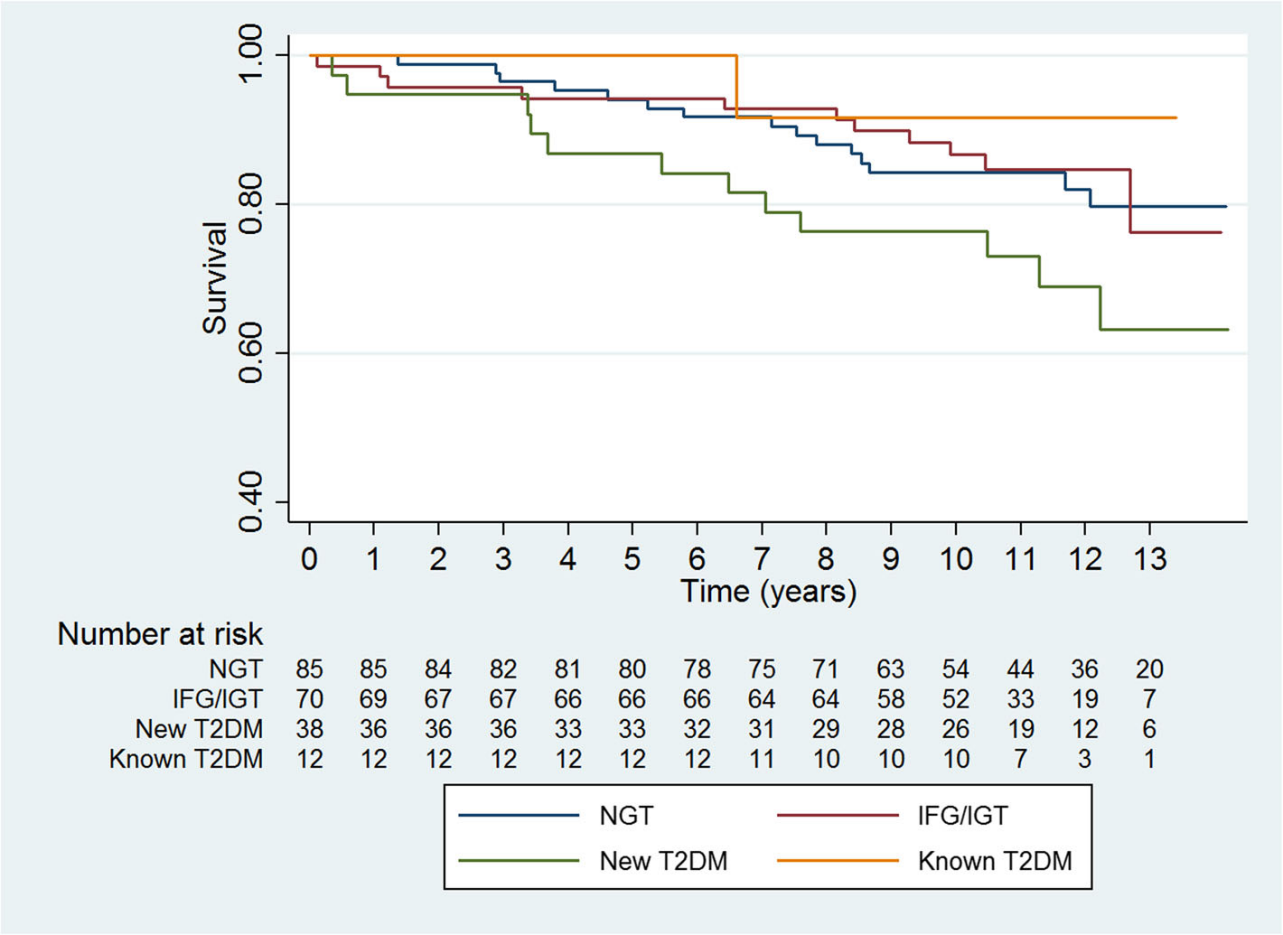
Kaplan-Meier curve shows survival according to all-cause mortality in 205 patients admitted with an acute myocardial infarction up to 14 years of follow-up. Patients without known type 2 diabetes mellitus were stratified according to an oral glucose tolerance test performed at time of admission

Patients were followed up to 14 years with a mean follow-up time of 10 years until end of follow-up or death. Kaplan-Meier curve showed (Fig. 3) event rates of all-cause mortality in relation to dysglycemia. Patients with new T2DM had the highest event rates of all-cause mortality compared to the other OGTT groups. Patients with NGT had higher event rates of all-cause mortality compared to patients with known T2DM, but comparable event rates of all-cause mortality as patients with IFG/IGT. Log rank test showed no difference between OGTT groups (*P* = 0.16).

In cox proportional regression model (glucometabolic status by OGTT (NGT, IFG/IGT, new T2DM) and known T2DM), higher HR of MACE was only observed in patients with new T2DM (HR 1.93 [95% CI 0.90-4.12]) compared to patients with NGT, however not statistically significant (Table 5F). Patients with IFG/IGT and known T2DM had lower HR of MACE compared to patients with NGT, however not statistically significant.

Log rank test showed no significant difference in patients with NGT and IFG/IGT (*P* = 0.82). Patients with NGT and IFG/IGT were then pooled and used in a modified cox proportional hazard model. Patients with new T2DM (HR 2.00 [95% CI 1.01-3.97]) showed a significantly increased HR compared to patients with NGT/IFG/IGT. Patients with known T2DM (HR 0.49 [95% CI 0.07-3.59]) showed an insignificantly reduced HR compared to patients with NGT/IFG/IGT.

## Discussion

To our knowledge, this observational study is the first to investigate the association of dysglycemia evaluated by a 2-hour OGTT on LVM in normotensive patients admitted with a first MI. Our data demonstrates a strong association between increasing dysglycemia and increased LVM. Dysglycemia by OGTT was able to predict an increase in LVM independently of other cardiovascular risk factors, however only in the OGTT-groups with more pronounced glucometabolic disturbances. The causal effect of dysglycemia on LVM was supported by the observed associations between increasing levels of HbA1c and increased LVM but also increasing levels of fasting glucose and increased LVM. Furthermore, patients with new T2DM did not have significantly increased hazard of neither MACE nor all-cause mortality, despite a noticeably increased event rate in patients with new T2DM.

### Dysglycemia and LVM

Results from a study by Al-Daydamony MM et al. [7] investigated the impact of metabolic syndrome (MetS) on LVM in a population with and without hypertension and their findings support our results. They found a significantly positive correlation between increased LVM and fasting blood glucose (r = 0.52, *P* <  0.0001), but also a significantly positive correlation between LVM and HbA1c (r = 0.42, *P* <  0.0001). In their logistic regression model, only triglycerides, fasting blood glucose levels, HbA1c and HDL cholesterols, remained independent predictors of increased LVM. Associations between dysglycemia and LVM were observed despite the differences in study population.

Dysglycemia, insulin resistance and hyperinsulinemia have been investigated thoroughly [21, 22], but the findings were not consistent. Galvan et al. [21] investigated the association between insulin resistance and hyperinsulinemia in relation to LVM in a population of normoglycemic obese and hypertensive patients, however they did not find any association. Yasunari et al. [22] investigated the impact of dysglycemia on cellular level and stated, that dysglycemia may be linked to increased proliferation and hypertrophy through increased phospholipase D and protein kinase c pathways. Our study was not suited for evaluation of the cellular mechanisms in increased LVM, but altered glucometabolic pathways due to dysglycemia and compensatory hyperinsulinemia are plausible. Patients with hypertension were excluded in this study, but a linear trend was still observed in LVM, when stratified for dysglycemia (Fig. 1).

The impact of insulin on left ventricle in hypertensive older subjects was investigated with an OGTT in a study by Scuteri et al. [23]. They concluded that hyperinsulinemia might be associated with an increased LVM. Furthermore, Rutter et al. [24] investigated the impact of glucose intolerance and insulin resistance on cardiac structure and function in the large Framingham study. The main results showed that glucose intolerance was more strongly associated with increased LVM in women compared to men. Also insulin resistance was associated with increased LVM in women alone, but this was largely explained for by obesity. Men had higher LVM compared to women, however not found statistically significant. No analysis was performed to evaluate effect modifications of sex in this study. It was possible to observe the impact of dysglycemia on LVM, when also adjusting for sex in our study.

Jørgensen et al. [25] investigated the impact of T2DM on cardiac structures in patients without coronary heart disease and compared them to an age- and sexmatched control group. They concluded that diabetes duration had a significant impact on cardiac function including increased LVM due to increasing wall thickness. We did not have any data on diabetes duration, but our data also showed a significantly positive trend with increased septal wall thickness. No significant trend regarding the posterior wall thickness was detected.

### The impact of metabolic syndrome on left ventricle mass

MetS is difficult to ignore in all these studies, but it is also complex to assess in terms of the additive effect on LVM. Studies have explored the role of dysglycemia and MetS on cardiac structure and function. A study by Hwang et al. [26] investigated the impact of insulin resistance and MetS. They found insulin resistance to be associated with abnormal cardiac function and structure independent of age, blood pressure and glucose intolerance. In another study by Brownell et al. [27], they investigated the impact of obesity on cardiac structure and function in patients after bariatric surgery. They found patients with BMI ≥ 50 to be independently associated with left ventricle hypertrophy, however no independent association with hypertension and diabetes. A similar study by Ippisch et al. [28] investigated the changes in cardiac structures and function before and after bariatric surgery, and they found significant changes in LVM after bariatric surgery, implying that weight loss indirectly could have an impact on cardiac structure. There was no difference in BMI across OGTT-groups in our study and it was also taken into account in the statistical model.

### Hyperinsulinemia as a potential cause of increased left ventricle mass

Stakos et al. [29] investigated the impact of glycosylated hemoglobin, fasting glucose, 2-hour OGTT and insulin sensitivity index on LVM in a population consisting of non-diabetic individuals with insulin resistance. In a multivariate logistic regression model, only glycosylated hemoglobin predicted increase in LVM independently of other glucometabolic confounders. A study by Shah et al. [30] investigated the impact of central obesity in terms of increased waist-to-hip-ratio and insulin resistance on cardiac remodeling in non-diabetic individuals. Patients with impaired fasting glucose had significantly higher levels of insulin and more pronounced insulin resistance compared to patients with normoglycemic status. Patients with impaired fasting glucose also had significantly increased LVM compared to patients with normoglycemic status. All of these findings results remained statistically significant in multivariate regression analysis. They concluded that central obesity and insulin resistance were associated with LVM across body mass index.

Other studies [31, 32] have stated, that body mass had an impact on LVM. However a study by Ebinc et al. [33] stated, that insulin resistance and insulin were not associated with left ventricle hypertrophy in a population of older healthy population, but obesity seemed to be an independent risk factor for left ventricle hypertrophy.

Sundstrom et al. [34] investigated the impact of metabolic syndrome and insulin resistance on left ventricular geometry independent of blood pressure in older healthy individuals without severe cardiac disease. LVM was increased in patients with metabolic syndrome and in some subgroups of normo- and hypertensive patients. The authors suggested a potential causal effect of insulin on LVM through different enzymatic pathways (protein kinase C). Furthermore, hyperglycemia itself, was proposed as a mediator of increased LVM throughout different growth-factors (β-1, phosphatidylinositol 3-kinase, protein kinase β). Finally, insulin was also mentioned as a mediator of the neurohormonal system, which could have an impact on the sympathetic nervous system and thereby have an impact on the pressure response in relation to the angiotensin-related pathways. Inflammation, volume-load and dyslipidemia were also mentioned as possible confounders to have an impact on LVM. They concluded, that insulin resistance and metabolic syndrome were related to increase in LVM despite blood pressure.

The literature on dysglycemia and LVM is not consistent, but insulin resistance and hyperinsulinemia seems to have an impact on LVM, but not independently of body mass and other confounders as part of multifactorial causes. Hyperinsulinemia is still thought to have an impact on LVM throughout unknown mechanisms.

### Dysglycemia by an oral glucose tolerance test

In this study OGTT was performed shortly after admission with MI. A study by Ye et al. [35] showed a similar diagnostic accuracy of an OGTT in patients with and without MI. It is therefore reasonable to assume, that dysglycemia evaluated shortly after the MI, is a reliable tool to risk-stratify.

There is a particular focus on detecting dysglycemia in patients with cardiovascular disease [36]. It is recommended to use fasting glucose values, 2-hour glucose values and HbA1c. There is no strict consensus regarding investigation of dysglycemia, but rather some recommendations which are then used differently worldwide.

Lopez-Lopez et al. [37] investigated the effect of simultaneously measuring HbA1c, fasting glucose values and a 2-hour glucose values in detecting new T2DM. They concluded that measuring both fasting and 2-hour glucose values showed the highest proportion of patients with new T2DM. Another study by Marini et al. [38] recommended the use of both fasting glucose values and 2-hour glucose values. The value of 1-hour glucose values instead of 2-hour glucose values, have also been investigated by Paddock et al. [39]. They stated that a 1-hour OGTT is as effective as a 2-hour OGTT in detecting T2DM. Data on fasting glucose and 2-hour OGTT were only available on these patients and should be comparable to dysglycemia determined by a 1-hour OGTT. The categorization of patients with dysglycemia in our study therefore seems applicable.

### Dysglycemia, major adverse cardiovascular events and all-cause mortality

Two recent studies [40, 41] have shown worse prognosis in patients admitted with MI and dysglycemia. In the study by George A et al. [40], patients with MI and no history of T2DM were examined. Age, new T2DM and IGT were significant independent predictors of major adverse cardiovascular events in the multivariate cox proportional hazard regression model, when also adjusting for other cardiovascular risk factors. In the study by Ritsinger V et al. [41], patients with MI and dysglycemia were compared to patients without MI but dysglycemia. Age and dysglycemia were significant independent predictors of major cardiovascular event, but only tested in univariate cox regression model. In this study, increased dysglycemia was not significantly associated with neither MACE nor all-cause mortality.

Patients with new T2DM had increased HR of both MACE and all-cause mortality compared to patients with NGT, however these findings were not statistically significant. Patients with new T2DM only had a significantly increased HR of all-cause mortality in the modified cox proportional hazard model (NGT/IFG/IGT, new T2DM and known T2DM), whereas patients with new T2DM had insignificantly increased HR of MACE compared to patients with NGT/IFG/IGT.

Overall, patients with either IFG/IGT or known T2DM had lower HR of both MACE and all-cause mortality compared to patients with NGT. Patients in this study included patients with a first MI without hypertension, and therefore considered as a low risk-group compared to patients with more than one MI and/or receiving antihypertensive medication.

A previous study have shown, that patients admitted with a MI and dysglycemia evaluated by either HbA1c or an OGTT have worse prognosis during long-term follow-up [42]. However, only normotensive patients with new T2DM seemed to have higher event rates of MACE and all-cause mortality in this study.

To our best of knowledge, no studies have looked into the prognosis of dysglycemia in first time MI patients without hypertension. Patients with NGT and IFG/IGT had the same event rate of MACE and all-cause mortality during long-term follow-up. This could be partly explained by the combination of relatively small OGTT-subgroups and relatively low numbers of events throughout follow-up, which could have reduced the power in this study.

In this study, dysglycemia was not able to significantly predict MACE during long-term follow-up in this low-risk population of normotensive patients admitted with a first MI. A significantly increased HR of all-cause mortality was only detected in patients with new T2DM compared to patients with NGT/IFG/IGT.

### Study limitations

A limitation in this study is the selection bias due to exclusion of patients receiving any antihypertensive medication and the missing data on echocardiograms. Patients with T2DM are more likely to receive antihypertensive medication due to higher comorbidity and the proportion of missing data on LVM was not evenly distributed in all of the four sub studies, which could be a potential selection bias in this study. No data on infarction size was available, but there were no significant echocardiographic differences in baseline characteristics. Echocardiographic measurements were obtained from 4 different studies, however no assessment of inter-observer variability was possible to perform. Data on insulin levels would have strengthened our data, but these data was not available.

### Perspective

Our study adds to the literature regarding the association between dysglycemia and LVM, even though the population consisted of normotensive patients with a first MI. The specific effect of MetS on increased LVM could not be properly determined, but dysglycemia still seems to have a significant impact. Patients with MetS should therefore have their cardiovascular risk factors reduced and accordingly having their dysglycemia improved. Our results may also be seen in perspective of the results from the EMPA-HEART trial, which is a follow-up to the EMPA-REG OUTCOME trial, where inhibition of sodium-glucose transport protein 2 resulted in salutary effects on LV remodelling [43].

## Conclusions

Dysglycemia is associated with significantly increased LVM independently of other cardiovascular risk factors in a population of normotensive patients with a first myocardial infarction. Higher prevalence of left ventricle hypertrophy is seen in patients with higher degrees of dysglycemia. The causal anabolic effect of hyperinsulinemia associated with dysglycemia by a 2-hour OGTT on LVM is most distinct in patients with new T2DM and known T2DM. Patients with new T2DM have higher event rates of both MACE and all-cause mortality compared to patients with either NGT or IFG/IGT. A significantly increased HR of all-cause mortality is detected in patients with new T2DM only, when compared to patients with NGT/IFG/IGT. Overall, dysglycemia is not an independent predictor of neither MACE nor all-cause mortality during long-term follow-up in normotensive patients with a MI.

## Additional file

Additional file 1: STROBE diagram. (DOCX 27 kb)

## Abbreviations

AGT: Abnormal glucose tolerance test
BMI: Body mass index
BSA: Body surface area
CABG: Coronary artery bypass graft
HR: Hazard Ratio
IFG: Impaired fasting glucose
IGT: Impaired glucose tolerance
LVH: Left ventricle hypertrophy
LVM: Left ventricle mass
MACE: Major adverse cardiovascular event
MetS: Metabolic syndrome
MI: Myocardial infarction
New T2DM: Newly detected type 2 diabetes mellitus
NSTEMI: Non-ST-segment elevation myocardial infarction
OGTT: Oral glucose tolerance test
SA: Stable angina
STEMI: ST-segment elevation myocardial infarction
T2DM: Type 2 diabetes mellitus
