# Supplementary material for: Dysglycemia and increased left ventricle mass in normotensive patients admitted with a first myocardial infarction: prognostic implications of dysglycemia during 14 years of follow-up
Source: BMC Cardiovasc Disord. 2019 May 2;19:103. doi: 10.1186/s12872-019-1084-5 (PMC6498536; doi:10.1186/s12872-019-1084-5)
Supplement: Supplementary file 1 — STROBE diagram. (DOCX 27 kb) [file 12872_2019_1084_MOESM1_ESM.docx]

**Additional file 1**

Included (n= 205)

Assessed for eligibility (n= 656)

STROBE diagram

Excluded (n= 451)

-  Previous MI n= 66 (15%)

-  Hypertension n= 274 (61%)

-  Missing data n= 111 (24%)

- LVM n= 109 (98%)

Study 1: 2% missing data on LVM [15]

Study 2: 42% missing data on LVM [16]

Study 3: 66% missing data on LVM [17]

Study 4: 23% missing data on LVM [18]

- OGTT n= 1 (1%)

- BSA n= 1 (1%)

MI, myocardial infarction. LVM, left ventricle mass. OGTT, oral glucose tolerance test. BSA, body surface area.
